# Burden of diabetic retinopathy in mainland China: Protocol for an updated systematic review and meta-analysis of prevalence and risk factors to identify prevention policies

**DOI:** 10.1097/MD.0000000000013678

**Published:** 2018-12-14

**Authors:** Yifan Zhong, Jinyang Wu, Song Yue, Guisen Zhang, Lei Liu, Lei Chen

**Affiliations:** a Department of Ophthalmology, The First Hospital of China Medical University; b Hohhot Chao Ju Eye Hospital; c Public Health Service, The First Hospital of China Medical University; d Liaoning Diabetic Eye Center, Shenyang, Liaoning Province, China.

**Keywords:** burden, diabetic retinopathy, mainland China, meta-analysis, prevalence, risk factors

## Abstract

**Background:**

Diabetic retinopathy (DR) is the leading cause of vision loss in adults of working age. Although existing systematic reviews of the prevalence of DR for mainland China have been reported, but several studies have been newly reported. Further some potential factors for DR are still discrepant and inconclusive. The aim of current research is to identify relevant literature regarding the prevalence of DR and DR-related factors in mainland China. In addition, we will project the number of individuals affected with DR in mainland China in years 2030 and 2050.

**Methods:**

This systematic review will follow the Preferred Reporting Items for Systematic Reviews and Meta-Analyses (PRISMA) checklist. To identify publications in English or Chinese languages on the prevalence of DR in mainland China, a comprehensive step-by-step search of the PubMed, Embase, Web of Science, VIP Chinese Periodical, and China National Knowledge Infrastructure (CNKI) databases will be performed. Two reviewers will independently review the studies for eligibility according to the predefined inclusion and exclusion criteria. The quality of the included studies will be evaluated according to the National Health Institute Quality Assessment tool and Quality Assessment Manual. A random-effect model (DerSimonian-Laird method) will be used for the meta-analysis. Heterogeneity and publication bias among studies will be estimated by the *I*^2^ statistic and Begg funnel plot, respectively.

**Results:**

This systematic review study will provide an evidence of prevalence, risk factors, and national burden for DR in mainland China.

**Conclusion:**

The study will give an explicit evidence to provide preventative measures of DR.

PROSPERO registration number: CRD42018094565.

## Abstract 2

Supplemental Digital Content is available in the text

## 1 Introduction

According to a recent estimate by the Vision Loss Expert Group of the Global Burden of Disease Study, there were 216.6 million people with moderate or severe vision impairment (VI) in 2015, of which 36 million were blind. By 2020, the number of people with moderate or severe VI is anticipated to rise to 237.1 million, whereas the number of global population with blindness is anticipated to rise to 38.5 million.^[1]^ Till date, diabetic retinopathy (DR) is one of the most common causes of VI in adults aged from 20 to 74.^[2]^

A recent nationally representative cross-sectional survey in 2013 reported that the overall prevalence of diabetes mellitus (DM) was 10.9%, and that prediabetes was 35.7% among adults in mainland China.^[3]^ In 2015, the International Diabetes Federation estimated that China has the world's largest population of adults with DM, which continues to increase.^[4]^ DM increases the risk of serious VI and blindness from retinopathy.^[5]^ A previous meta-analysis, 1980 to 2008, revealed that the patients with diabetes account for 34.6% (95% confidence interval [CI]: 34.5–34.8%) of those with DR.^[6]^ Although many strategies are performed to prevent DR,^[7]^ it is more prevalent with increasing prevalence of DM, especially in China.^[8]^ China is vast in area, thus, it is very difficult to perform a nationally representative survey on the prevalence of DR. Our earlier meta-analysis has covered the studies on the prevalence of DR from mainland China which was published in 2012.^[9]^ These findings revealed that the pooled prevalence of any-DR, nonproliferative diabetic retinopathy (NPDR), and proliferative diabetic retinopathy (PDR) in general population was 1.3% (95% CI: 0.5–3.2%), 1.1% (95% CI: 0.6–2.1%), and 0.1% (95% CI: 0.1–0.3%), respectively, whereas it was 23% (95% CI: 17.8–29.2%), 19.1% (95% CI: 13.6–26.3%), and 2.8% (95% CI: 1.9–4.2%) among people with diabetes. The pooled prevalence of any-DR in rural population was higher than that in the urban population. Moreover, the prevalence of any-DR was higher in the Northern region compared with that of the Southern region. Recently, another systematic review and meta-analysis provided a comprehensive estimation of the prevalence of DR in China,^[10]^ but there is a lack of data on the prevalence of DR among different ethnic groups in Chinese.

So far, the risk factors for DR among Chinese have not been clear. Although recent meta-analysis investigated some factors for DR, other potential factors such as high-density lipoprotein cholesterol, low-density lipoprotein cholesterol, and diastolic blood pressure for DR are still discrepant and inconclusive among the Chinese population, which need to be systematically evaluated in an evidence-based fashion.^[10]^ Many previous studies found that triglyceride (TC) was an independent risk factor for DR among Chinese patients with diabetes,^[8,9,11]^ whereas other studies revealed that there was no significant association between the levels of TC and the presence of DR.^[12,13]^ A systematic review and meta-analysis of the risk factors for any-DR, NPDR, PDR, and vision-threatening diabetic retinopathy (VTDR) will thus provide important insights and assist ophthalmologists in preventing DR in the future. Accurate estimates of risk factors for DR are useful when establishing predictive model and it will be important in the near future when this model may be used for personalized estimates of disease risk.

With more attention paid to population ageing and DR epidemic, many new epidemic surveys have been performed in mainland China, and published in recent years.^[14–16]^ This provides an opportunity to conduct an updated meta-analysis on the prevalence of DR in mainland China and forecast the national burden of DR. Our meta-analytic study will address the following questions: The prevalence of DR in mainland China and disease burden for the years 2030 and 2050; The variation in the prevalence of DR among different ages, genders, and ethnic groups, together with area; and The pooled risk factors including molecular biomarkers for DR within Chinese.

## 2 Materials and methods

This systematic review protocol has been registered on PROSPERO (http://www.crd.york.ac.uk/PROSPERO/) under the number of CRD42018094565, and was performed in accordance with the Preferred Reporting Items for Systematic Reviews and Meta-analysis Protocol (PRISMA-P), which provides a standardized guide for performing systematic reviews and meta-analysis.^[17]^ In addition, the quality of the individual studies included in this systematic review will be evaluated independently by two reviewers using the National Health Institute Quality Assessment tool (https://www.nhlbi.nih.gov/health-pro/guidelines/indevelop/cardiovascular-risk-reduction/tools/cohort). Report of the clinical examination method or retina photos used to determine DR status, and grade of retinopathy using the Early Treatment Diabetic Retinopathy Study standard^[18]^ or International Clinical Diabetic Retinopathy Disease Severity Scale.^[19]^ The search strategy used in the previous report 2012 and the updated search strategy are shown in Table 1.

**Table 1:**
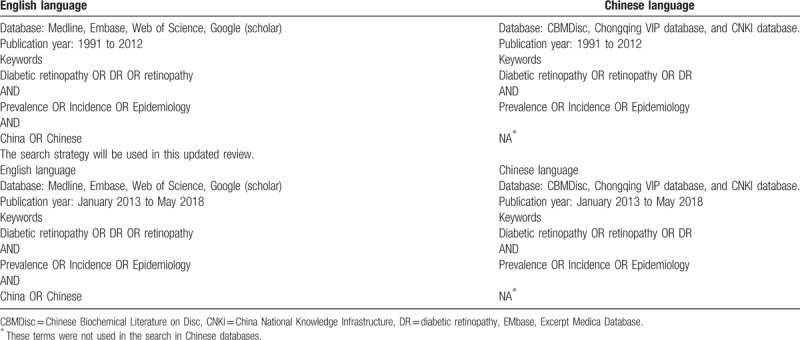
The search strategy used in the previous report and this review.

Prevalence will be estimated by using four cutoffs: any-DR, NPDR, PDR and VTDR. Any-DR will be defined as background retinopathy or more severe disease. VTDR, which requires prompt medical treatment to reduce the risk of visual impairment, will be defined as the presence of severe NPDR or PDR and/or diabetic macular edema (also called clinically significant macular edema).^[20]^

### 2.1 Inclusion and exclusion criteria

In this systematic review and meta-analysis, studies that report prevalence estimates of DR among people with diabetes in mainland China will be eligible for our study. Inclusion and exclusion criteria are shown in Table 2.

**Table 2:**
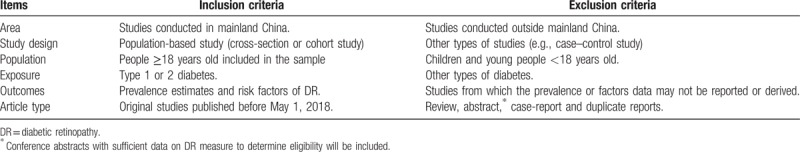
Inclusion and exclusion criteria.

### 2.2 Study selection

The search results will be reported to Microsoft Excel (Microsoft Corporation, Redmond, WA). Two reviewers will independently screen titles and abstracts, and then go to screen full-text manuscripts according to the eligibility criteria. In the absence of an amicable settlement of any disputes, the disputes shall be settled by one arbitrator who shall act as an amiable compositeur.

### 2.3 Data extraction

Two reviewers will extract the data from the included studies using a standardized form independently: Characteristics of including studies: first author, study design (cross-sectional or cohort study), year of publication, examiner for DR; Participants: sampling methods, sample size and response rate, characteristics of participants, such as age group, sex group, type of diabetes, location, duration of diabetes, and levels of glucose; DR identification: diagnostic tests and grading protocol; Results: overall number of any-DR cases and stratified number of cases by age, gender, and severity of retinopathy. The risk factors for both any-DR and VTDR reported in the included studies.

### 2.4 Assessment of risk of bias in included studies

It is very important to assess the risk of study bias when we perform and interpret systematic reviews of the literature. In this systematic review, the risk of including study bias will be assessed using an existing tool^[21]^ (Supplement 1). Interrater agreement was found to be high in this tool and it was easy to apply.

### 2.5 Data synthesis

In this systematic review and meta-analysis, extracted data will be presented in comprehensive tables, flowcharts, and graphs, accordingly. The prevalence estimates of DR extracted from including studies will be standardized to the Census Population of China 2010. Presuming that prevalence estimates are variable between different populations, thus, pooled prevalence estimates of any-DR, NPDR, PDR, and VTDR will be calculated by a random effect model in this meta-analysis. We will use forest plots to represent individual and pooled estimates of the prevalence. Quantitative assessment of heterogeneity between studies will be conducted using the *I*^2^ statistic.^[22]^ In addition, publication bias will be assessed by Begg's funnel plot.

The China's DR burden projection of disease for the years 2030 and 2050 will be projected according to the World Population Prospects.^[23]^

### 2.6 Subgroup analysis

In order to reduce the random variations between the estimates of the primary study, we will perform subgroup analysis based on the different types of participants (e.g., by age, gender, ethnicity), regions where the studies were conducted, and sample sizes of the studies.

### 2.7 Ethics and dissemination

Ethics approval is not required as this is a systematic review and meta-analysis using published data. We will report our findings of this systematic review and meta-analysis in a peer-reviewed journal in future.

## 3 Discussion

The burden of diabetes is increasing in China, as a microvascular complication: DR is becoming a common cause of VI and blindness in working-age population. In this updated systematic review and meta-analysis, we will include data regarding the prevalence of DR in other areas of mainland China. Moreover, this updated systematic review and meta-analysis will provide summarized data to establish national prevalence estimates and project the number of people with DR from 2030 to 2050 which will be a useful guide for national strategies to control DR. In addition, this meta-analysis, using pooled odds ratios to evaluate the factors for DR, will provide evidence-based policy and practice for Chinese people living with diabetes.

## Author contributions

**Data curation:** Yifan Zhong, Jinyang Wu.

**Formal analysis:** Song Yue, Lei Liu.

**Investigation:** Guisen Zhang, Lei Liu.

**Methodology:** Jinyang Wu, Lei Liu.

**Resources:** Yifan Zhong.

**Software:** Lei Liu.

**Supervision:** Lei Liu, Lei Chen.

**Validation:** Lei Liu, Lei Chen.

**Writing – original draft:** Yifan Zhong, Lei Liu.

**Writing – review & editing**: Lei Liu.

## Supplementary Material

Supplemental Digital Content
