# Supplementary material for: Burden of diabetic retinopathy in mainland China: Protocol for an updated systematic review and meta-analysis of prevalence and risk factors to identify prevention policies
Source: Medicine (Baltimore). 2018 Dec 14;97(50):e13678. doi: 10.1097/MD.0000000000013678 (PMC6320130; doi:10.1097/MD.0000000000013678)
Supplement: Supplemental Digital Content [file medi-97-e13678-s001.docx]

**Additional file 1: Quality assessment**

**External validity**

1. Was the sampling frame a true or close representation of the target population?

Yes: The study`s target population was a close representation of the national population.

No: The study`s target population was clearly NOT representative of the national population.

2. Are the study subjects described in detail regarding?

Yes: Age, gender or diabetes duration was reported along with a measure of diabetes control and the year(s) when the study was conducted.

No: One or more of the previous details was not described.

3. Was some form of consecutive, random selection used to select the sample, OR was a census undertaken?

Yes: Yes.

No: No or unclear.

4. Was the likelihood of non-response bias minimal?

Yes: Response >50%.

No: Unclear, not reported, or less 50%.

**Internal validity**

5. Were data collected directly from the subjects (as opposed to a proxy)?

Yes: All data were collected directly from the subjects.

No: In some instances, data were collected from a proxy.

6. Was an acceptable case definition used in the study?

Yes: Internationally acceptable (Early Treatment Diabetic Retinopathy Study Standard or International Clinical Diabetic Retinopathy Disease Severity Scale) taxonomy of diabetic retinopathy was used.

No: No clear definition of diabetic retinopathy, e.g. retinal changes, or the severity of cases was not described.

7. Was the study instrument that measured the parameter of interest shown to have validity and reliability?

Yes: Retinal photographs or fluorescein angiogram.

No: Fundoscopy or ophthalmoscopy alone.

8. Was the same mode of data collection used for all subjects?

Yes: The same mode of data collection was used for all subjects.

No: The same mode of data collection was NOT used for all subjects.

9. Was the length of the shortest prevalence period for the parameter of interest appropriate?

Yes: The shortest prevalence period for the parameter of interest was appropriate (e.g. point prevalence, one-week prevalence, one-year prevalence).

No: The shortest prevalence period for the parameter of interest was not appropriate (e.g. lifetime prevalence).

10. Were the numerator(s) and denominator(s) for the parameter of interest appropriate and confidence interval given?

Yes: Confidence interval given.

No: Confidence interval not given.

11. Was the sample size equal or greater than 200?

Yes.

No.
